# Efficacy of online communication partner training package for student healthcare professionals

**DOI:** 10.1111/1460-6984.12947

**Published:** 2023-09-03

**Authors:** Emma Power, Michelle C. Attard, Lucette E. Lanyon, Leanne Togher

**Affiliations:** ^1^ University of Technology Sydney Sydney NSW Australia; ^2^ Centre of Research Excellence in Aphasia Recovery and Rehabilitation La Trobe University Melbourne VIC Australia; ^3^ La Trobe University Melbourne VIC Australia; ^4^ The University of Sydney Sydney NSW Australia

**Keywords:** aphasia, clinical education, communication training, curriculum, healthcare, stroke

## Abstract

**Background:**

People with aphasia are vulnerable recipients of healthcare. The nature of the communicative environment and the communication disability can adversely impact access to timely and quality healthcare. Student healthcare professionals are often underprepared to interact successfully with people with aphasia and may benefit from communication partner training (CPT).

**Aims:**

To investigate the potential effectiveness and acceptability of a brief, two‐part introductory Supported Conversation for Adults with Aphasia (SCA™)‐based CPT package, delivered to a sample of students across a diverse range of healthcare disciplines.

**Methods & Procedures:**

A pre–post‐within group experimental design was used to investigate the potential effectiveness and acceptability of an online CPT package (50 minute module + 1 hour workshop) for healthcare students. The Aphasia Attitudes, Strategies and Knowledge (AASK) survey measured participants’ knowledge of aphasia, facilitative communication strategies and attitudes towards people with aphasia. Data were collected pre‐training, following the training module and following the workshop, and 6 weeks post‐training. Statistical analysis was conducted on the AASK data. In addition, participant feedback (ratings and open text responses) was collected after the workshop. Ratings were analysed descriptively, and thematic content analysis was used for open text responses.

**Outcomes & Results:**

236 participants completed the pre‐training AASK and 106 completed the AASK at subsequent time points. Statistically significant gains were demonstrated from pre‐ to post‐module completion. Between the end of the module and the end of the workshop, some gains were maintained and others showed further statistically significantly improvements. While all gains were not maintained at the 6‐week follow‐up, statistically significantly improvements from pre‐training scores remained evident. Student feedback was predominantly positive, with suggested improvements for training content and length.

**Conclusions & Implications:**

The results provide preliminary evidence that a brief, online CPT package can support student healthcare professionals’ knowledge and attitudes towards aphasia and communicating with people with aphasia. Online training was acceptable to students and feasible as an embedded or optional component of curriculum. Ongoing training (e.g., in the form of refresher sessions) and inclusion of a skills‐based component are recommended to maximize communication skill development.

**WHAT THIS PAPER ADDS:**

**What is already known on the subject**

Student healthcare professionals recognize the need to develop knowledge and skills to successfully support people with communication disability, such as aphasia, to participate effectively in their healthcare. Evidence in favour of online communication partner training for student healthcare professionals is currently limited.

**What this study adds to the existing knowledge**

This study demonstrates that a brief introductory online communication partner training program can be efficacious for improving knowledge and attitudes regarding communicating with people who have aphasia.

**What are the potential or actual clinical implications of this work?**

Students will likely need further ongoing refresher training with inclusion of practical components to develop and maintain the knowledge and skills required to be proficient communication partners with people with aphasia.

## INTRODUCTION

Aphasia is a pervasive communication disorder commonly occurring after stroke and other brain injury. The number of stroke survivors with aphasia is estimated at 30–35% in middle‐to‐high‐income countries such as Australia, Canada, Switzerland, the United States, the UK and Germany (Deloitte Access Economics, 2020; Engelter et al., 2006; Flowers et al., 2016; Lam & Wodchis, 2010). It can impact all communication modalities (Benson & Ardila, 1996)—verbal expression, verbal comprehension, reading, writing and gesturing—and have devastating impacts on life participation and quality of life (Cruice et al., 2006). People with aphasia are considered ‘communication vulnerable’ in medical settings. They are at increased risk of harmful adverse events and reduced health outcomes as a direct consequence of their communication impairment (Hemsley et al., 2016, 2019). People with aphasia require communication supports to ensure they can optimally engage in their healthcare (Beukelman et al., 2016). The World Health Organization's (WHO) International Classification of Functioning, Disability and Health (WHO, 2001) places an emphasis on the environment and the degree to which the environment functions as a barrier or facilitator to a specific activity and participation domain. Healthcare environments can be inaccessible for people with aphasia when healthcare provider knowledge, skills, attitudes and experience is inadequate to support their communication needs (O'Halloran et al., 2012) and this can disrupt usual healthcare provision (Carragher et al., 2021). Research has demonstrated that, for example, health professionals may control or limit patient interactions when the patient presents with aphasia (Hersh et al., 2016). Health professionals perceive interactions with people with aphasia to be time consuming, and as a consequence redirect conversations to family members thus impacting on the person with aphasia's ability participate in their own health decision making (Carragher et al., 2021). To reduce the environmental barriers to communication for people with aphasia we need to have education, training and a shift in ward culture (Carragher et al., 2021).

Communication partner training (CPT) provides important conversation partners (e.g., spouses, healthcare providers) with facilitative communication strategies directed at improving the quality of communicative exchanges and reducing communication breakdown (Simmons‐Mackie et al., 2010). CPT is recommended in international clinical practice guidelines to enhance the communicative environments of people with aphasia (Power et al., 2015; Royal College of Physicians Intercollegiate Stroke Working Party, 2016). The efficacy of face‐to‐face CPT for aphasia is well established (Simmons‐Mackie et al., 2016, 2010; Tessier et al., 2020), particularly for healthcare professionals (Heard et al., 2017; Horton et al., 2016; Jensen et al., 2015; van Rijssen et al., 2019; Welsh & Szabo, 2011). Further, not only does CPT enhance communicative exchanges and experiences (Finch et al., 2018), it also has the potential to prevent negative exchanges and experiences when an unskilled communication partner is repeatedly exposed to people with aphasia (Kagan et al., 2018). Additionally, there is an increasing evidence base for online learning and hybrid aphasia CPT programs, with Heard et al. (2017) finding no significant differences in health professionals’ confidence and knowledge of aphasia after a CPT delivered face to face or through e‐learning. The authors concluded that due to the feasibility and scalability advantages of online delivery formats, future research should include online modes of delivery.

While the majority of aphasia CPT research has been conducted with qualified health professionals, there is a developing evidence base for the benefits of providing aphasia CPT to student health professionals from a variety of disciplines and in a range of delivery formats (Cameron et al., 2015; Doherty & Lay, 2019; Finch et al., 2018, 2020; Legg et al., 2005; Power et al., 2020). CPT can improve skills related to knowledge of aphasia; knowledge of communication strategies; and confidence in interacting with people with aphasia when delivered face to face for speech pathology, physiotherapy, occupational therapy and medical students (Cameron et al., 2015; Finch et al., 2018; Legg et al., 2005). A smaller number of studies have demonstrated that knowledge, confidence and possibly skills can improve after a combination of face to face and telehealth aphasia CPT for speech pathology students (Finch et al., 2020) or face to face and online flipped learning approaches for occupational therapy students (Doherty & Lay, 2019). Further, Power et al. (2020) found that for 30 occupational therapy students, there was no significant difference between face‐to‐face didactic and self‐directed online learning CPT delivery modes in increasing knowledge of aphasia; knowledge of communication strategies, and confidence in interacting with people with aphasia. Some limitations in these studies include small participant numbers (*n* = 6–38) with one exception (Cameron et al., 2018; *n* = 77), however this study provided qualitative outcomes on perceived benefits in a smaller subset of students without any quantitative efficacy data. Most studies reported on a single healthcare discipline and had differing aims including comparing delivery modality (Power et al., 2020), feedback effects (Finch et al., 2020) and CPT components (Legg et al., 2005). Several studies were restricted to face‐to‐face‐only formats (Cameron et al., 2018, 2015; Finch et al., 2018; Legg et al., 2005), or did not include a more active learning component such as roleplays (Power et al., 2020). When a study did include conversations with people with aphasia and active application of the lecture learning, the session was brief (10–15 minutes) with 5 minutes of feedback, often in groups of up to three students (Finch et al., 2018, 2020). Other studies have targeted broader communication disability training for various neurological populations (e.g., Parkinson's disease) (Burns et al., 2017; Forsgren et al., 2017; Saldert et al., 2016). None of the studies above contained a follow‐up measurement phase to establish maintenance of outcomes.

Explicit provision of CPT may be critical. An Australian survey of student speech–language pathologists who had received aphasia‐related coursework focused on theory rather than application of knowledge, found that students were not confident in their ability to communicate with people with aphasia (Finch et al., 2013). This finding is also likely to be the case for students in other healthcare disciplines that may also have less theoretical training on the topic. Therefore, to address several of the issues in the current evidence base, we aimed to establish whether a fully online training format is efficacious. The training format consists of a self‐directed online learning module and a videoconferencing‐based active learning workshop. Our goal was to determine if this training format can improve knowledge of aphasia and communication strategies, attitudes, and confidence for a larger number of students studying a range of healthcare disciplines. We also wished to determine if outcomes were maintained over a 6‐week follow‐up period. If shown to have efficacy, an online and video conferencing‐based CPT program could be both flexible and potentially scalable, fitting into contemporary university curriculum pedagogy where learning comprises both of asynchronous and self‐directed elements, accomplished through self‐motivation and independent learning combined with more facilitated active learning methods (Mukhalalati & Taylor, 2019).

## AIMS

This study aimed to investigate the potential effectiveness and acceptability of a brief, two‐part introductory Supported Conversations for Adults with Aphasia (SCA™) (Kagan, 1998)‐based CPT package delivered to a sample of students across a diverse range of healthcare disciplines. The first component comprised of an online 50‐minute self‐directed training module, and the second component consisted of an online 1‐hour workshop led by a trained speech pathologist.

The research questions were:

1. For a sample of multidisciplinary student healthcare professionals, does the two‐part CPT package improve outcomes regarding (1) knowledge of aphasia, (2) knowledge of facilitative communication strategies for engaging with people with aphasia, and (3) attitudes towards communicating with people with and without aphasia? (Potential efficacy).
2. What are students’ perceptions of the two‐part CPT package? (Acceptability).

Hypotheses:

We hypothesized for question 1 that:

- completion of both the training components would significantly improve outcomes 1–3;
- completion of the workshop (training Part 2) would result in further significant improvement in outcomes 1–3 beyond those reported following the module (training Part 1); and
- improvements in outcomes 1–3 would be maintained 6 weeks after the workshop (training Part 2).

For question 2, we predicted that overall, the online nature of the program (including workshops) and the content would be acceptable to students, and that they would suggest some elements that could be improved in the next iteration for both the delivery and content of the training.

## METHODS

### Study design

A pre–post‐within‐group experimental design was used to explore CPT outcomes before training (Time 1), following training Part 1—online module (Time 2), following training Part 2—online workshop (Time 3), and 6 weeks following training Part 2 (Time 4). This study received approval from La Trobe University's Human Ethics Committee (reference number HEC20165).

### Participants

**FIGURE 1:**
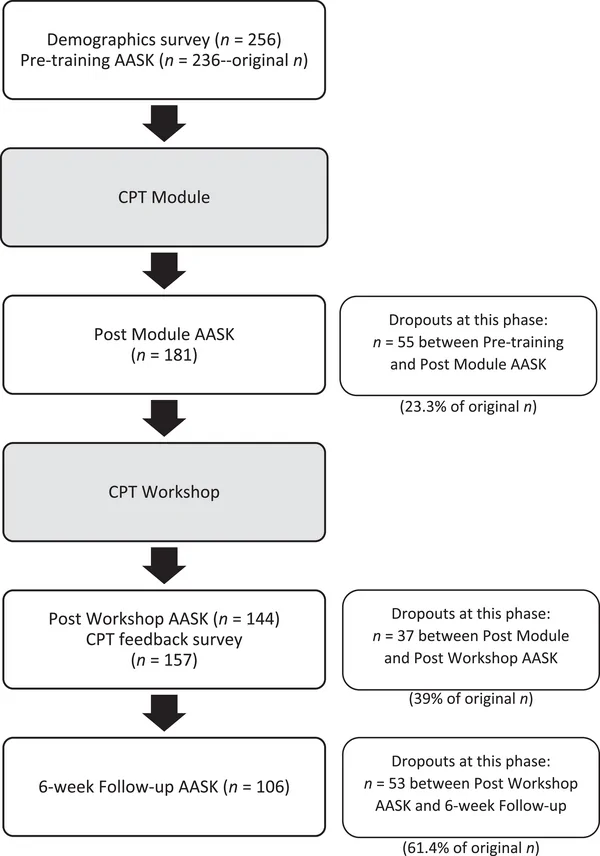
Study design and participant flow* through data collection and training phases.

Undergraduate and postgraduate student healthcare professionals aged 18 or over were recruited from La Trobe University over 3 months. Faculty staff from a range of disciplines were approached with the opportunity to embed the CPT into their course, or to offer it as an extracurricular activity. Participants were identified and recruited via digital correspondence provided by unit coordinators and the second author via the university's learning management system. Students were given the opportunity to self‐select to participate in the research. Figure 1 depicts the study's design and participant flow. Participant demographic characteristics are summarized in Table 1.

**TABLE 1:** Participant sample (*n* = 236) demographics.

Age (years): Mean (SD); Range	26 (9.97); 18–66						
Previous tertiary study undertaken	No: *n* = 119 (50.4%); Yes: *n* = 117 (49.6%)						
Previous exposure to aphasia and/or communication disability	‘No’, *n*, %	‘Yes’, *n*, %	Nature of exposure for ‘Yes’				
			(a) Family/friends	(b) Volunteering	(c) Own disability	(d) Combination of (a), (b) and/or (c)	(e) Unclear^d^
Previous personal exposure to aphasia	211 (89.4%)	25 (10.6%)	16	6	1	0	2
Previous personal exposure to communication disability	170 (72%)	66 (28%)	28	14	1	2	21
			**(a) Paid work**	**(b) University placements**	**(c) University coursework**	**(d) Combination of (b) and (c)**	**(e) Unclear^d^**
Previous professional exposure to aphasia			24	16	6	2	5
Previous professional exposure to communication disability	155 (65.7%)	81 (34.3%)	42	11	3	5	19
					*N* by program type		

					Undergraduate (year level)				Postgraduate (first and second year combined)		
Degree type	Relationship of training to coursework^a^	*N* potential ‘yield’	*N* Recruited, % of potential ‘yield’	% of *N* sample	1	2	3	4	–	*n* completed Time 1 to Time 4	% of n sample
Occupational therapy	Optional extra	358	87, 24%	37%	15	18	44	10	0	47	44
Physiotherapy	Optional extra	About 40 x year = 160	31, 19%	13%	7	6	8	10	0	17	16
Public health unit of study^b^	Embedded	100	27, 27%	11%	1	24	1	1	0	6	6
Art therapy	Optional extra	24	20, 83%	8%	n.a.				20	8	8
Clinical neuropsychology	Embedded	16	12, 75%	5%	n.a.				12	6	6
Clinical psychology	Embedded	24	10, 42%	4%	n.a.				10	2	2
Sports and exercise science	Embedded	About 40	9, about 23%	4%	1	0	8	0	0	6	6
Disability practice	Optional extra	About 80	17, about 21%	7%	15	0	0	0	2	3	3
Speech pathology	Embedded	64	9, 14%	4%	9	0	0	0	0	4	4
Orthoptics	Embedded	30	7, 23%	3%	7	0	0	0	0	3	3
Professional psychology	Embedded	8	4, 50%	2%	n.a.				4	2	2
Dietetics	Embedded	6^c^	3, 50%	1%	0	0	0	3	0	2	2
Total by degree/unit of study type	4 Optional; 8 embedded	About 622 optional; about 223 embedded	236 (155 Optional; 81 embedded)	66% Optional; 34% embedded	55	48	61	24	49	106	100

*Notes*:

^a^ Optional extra = students were invited to participate in the training as a supplement to their existing coursework, with optional research participation; Embedded = the training was embedded into students’ current coursework for a unit of study (completion was required), with optional research participation.

^b^ Students receiving this unit of study were undertaking the following bachelor's degrees: rehabilitation counselling, health sciences, biomedical science and double degrees (e.g., psychology and genetics).

^c^ Based on the remaining students available after version 1 was trailed (version 1: original potential yield 41, *n* = 16 recruited).

^d^ Unclear = only nature of communication deficit(s) indicated.

### Materials

#### Aphasia CPT content

The digital aphasia CPT package was entitled, ‘Communicating with People with Aphasia in Healthcare Contexts’ (Power et al., 2020). Based on SCA™ (Kagan, 1998), content areas comprised: (1) knowledge of aphasia and how aphasia can impact healthcare; (2) facilitative strategies for communicating with people with aphasia; and (3) attitudes towards communicating with people with aphasia. The facilitative strategies address the SCA™ tenets ‘acknowledging competence’ (creating respectful interactions) and ‘revealing competence’ (supporting people with aphasia to understand and express themselves in interactions) (Kagan, 1998). Consistent with earlier materials (Power et al., 2020), Part 1 of the package comprised a 50‐minute self‐directed online module. This was augmented with Part 2, a 1‐hour online group workshop developed for the present study, delivered over the teleconferencing platform Zoom and aimed to reinforce the online module with discussion and roleplay. The workshop had three primary goals: (1) to reinforce understanding of aphasia and how it can affect access to healthcare; (2) to reinforce understanding of strategies that can facilitate communication with people with aphasia and other communication disorders; and (3) to practice multimodal communication. In addition, students were encouraged to come to the workshop with specific questions about their learning and experiences. Over the 50‐minute workshop students participated in whole group and small group activities in which they completed role plays; reflected on communication in various health work settings; observed video‐based interactions between individuals with aphasia and health practitioners; and discussed examples from the observed interactions of effective communication techniques, acknowledging and revealing competence from the online module. No handouts or manuals were provided to participants.

### Outcome measures

For question 1 (potential effectiveness), participants completed the Aphasia Attitudes, Strategies and Knowledge (AASK) survey (Power et al., 2021) (see below) in digital form as the primary outcome measure at four time points: before training (Time 1), after the module (Time 2), after the workshop (Time 3), and 6 weeks after the workshop (Time 4).

#### Aphasia attitudes, strategies and knowledge (AASK) survey (Power et al., 2021)

The AASK (Power et al., 2021) (see Appendix A for question items and original answer guide and supplementary file for expanded scoring guidance) is an 11‐item survey constructively aligned with the content and learning objectives of SCA™‐based aphasia CPT. It examines participant knowledge of features of aphasia and its impact on access to healthcare services (Section 1). Section 2 examines participant knowledge of facilitative communication strategies (to acknowledge and reveal competence). Additionally, Section 3 examines levels of comfort and confidence with the prospect of communicating with people with and without aphasia. The AASK survey has strong test–retest, inter‐ and intra‐rater reliability (Power et al., 2021). Participants were instructed not to refer to any materials to assist with their responses during AASK survey completion. At Times 2–4, participants were asked to document any review/information‐seeking behaviours beyond the training that they had engaged in.

#### Participant feedback survey

**FIGURE E1:**
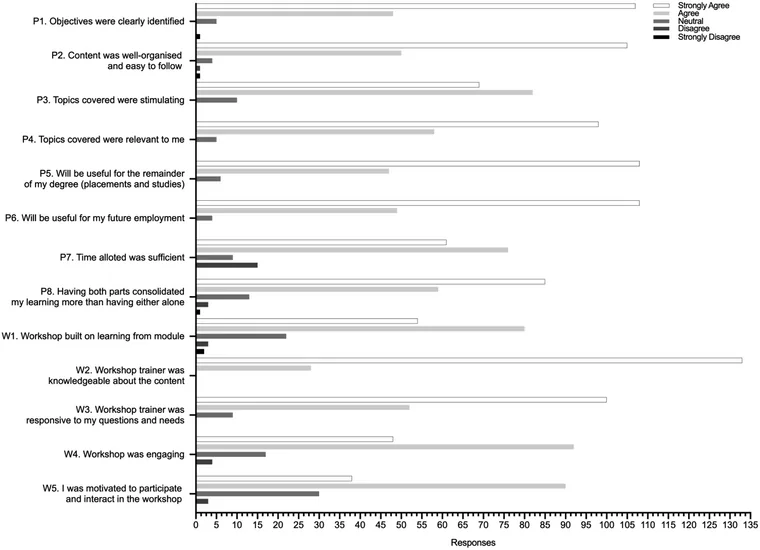
Feedback survey responses to Likert‐scale items. Note: P = items relating to the training package as a whole; W = workshop‐specific item. The inclusion of an additional training component has extended the application of the AASK, and we encourage interested readers to contact the last author for a copy of a comprehensive scoring guide developed as part of the rating process for the present study.

For question 2 (Acceptability), following completion of the training (Time 3), participants were invited to complete a 5‐minute digital feedback survey (see Figure E1 in Appendix E). The quantitative component comprised 13 Likert‐scale items relating to the training package as a whole, and five items relating specifically to the workshop, with response options ranging from strongly disagree to strongly agree (5 points). There were also three open‐ended response questions (addressing what was *most valuable/meaningful* about the package, what could be *changed/improved*, and any *other comments* expanding on the 13 Likert‐scale items).

### Procedure

The design and reporting of the intervention components of the study and training fidelity were based on the TIDieR checklist (Hoffmann et al., 2014) and are outlined in Table B1 in Appendix B.

All ratings were conducted with raters who were at all times blind to the randomized survey time points. Approximately 10% of a random sample of responses was rated by raters 1 and 2 (authors 2 and 3) with ratings then discussed in a consensus process between raters. When required, in order to further safeguard scoring accuracy, there was additional consultation with the first author to ensure consistency with the original AASK scoring system and agreed annotations. Rater 1 then used this amended scoring criteria and rated approximately 20% of the responses including rerating the initial 10% of samples. Rater 2 rated the remaining 80% of responses against the same criteria. Again, consensus discussions were held between Raters 1 and 2 if they were unsure about an answer, with any disagreements on final ratings resolved with the first author.

#### Data analysis

To establish our sample size, we calculated the sample size required using G*Power 3.1.9. (Faul et al., 2007), to achieve 0.8 power given the earlier (Power et al., 2021) effect size 0.71, alpha = 0.05, degrees of freedom 2. This calculation indicated a sample size of 20 was required. We also calculated the sample size for a parametric analysis (repeated measures multivariate analysis of variance (MANOVA) for a 1 group × 4 time point interaction for the present study) using a low‐to‐moderate effect size of 0.3, alpha = 0.05, correlation among repeated measures as moderate (0.3), and power of 0.8. This indicated a sample size of 26 was required. Therefore, accounting for a 15% attrition rate at each time point 2–4, approximately 40 participants were required.

Demographic and AASK survey data was analysed using the software package SPSS (version 26).

For question 1 (Potential effectiveness) we completed descriptive and inferential statistics on the AASK data. For the repeated measures MANOVA, Mauchley's test of sphericity was used to test the assumption that the error covariance matrix of the orthonormalized transformed dependent variables was proportional to an identity matrix. Using the conservative Lower‐bound Epsilon measure of sphericity, none of the dependent variables violated the assumption. SPSS packages do not have inbuilt multivariate tests of normality. Despite this, ANOVA designs are usually robust to violations of normality (Keselman et al., 1980; Stevens, 2009), so formal multivariate tests of normality were not performed. Instead, in line with accepted practice, each of the dependent variables were checked for outliers and eye‐balled for normality using skewness and kurtosis values, histograms, and Normal Q‐Q plots. None of the variables had major deviations from normal. To address question 1, a repeated‐measures MANOVA was used to compare the group‐level AASK scores (for Section 1, Section 2, Section 3A and Section 3B) across the four data collection phases. The alpha level was set at 0.05. The partial Eta squared (*η* ^2^) effect size for the MANOVA and Cohen's *d* effect size for post‐hoc pairwise comparisons are reported and are interpreted as follows: *η* ^2^: 0.01 = small; 0.06 = medium, 0.14 = large; Cohen's *d*: 0.2 = small, 0.5 = medium, 0.8 = large (Cohen, 1988).

For question 2 (Acceptability), participant feedback from the 13‐item survey data (Likert scale) was analysed using descriptive statistics. Open‐ended responses were analysed using an inductive qualitative content analysis (Sandelowski, 2000). Two researchers collaboratively developed relevant categories and iteratively refined categories and illustrative quotes through discussion into a final synthesized table (see Appendix D).

## OUTCOMES AND RESULTS

### Participant demographics

After completing the demographics form, 236 participants completed the pre‐AASK survey (Time 1). Participants’ mean age was 26 years and represented 12 ‘degree groups’ (Table 1). The majority of cohorts received the training as an embedded component of their coursework and reported no prior exposure to aphasia (*n* = 211, 89.4%) and/or other communication disability (*n* = 170, 72%). Prior exposure was reported by *n* = 66 (28%), primarily through paid work and family/friends. One hundred and six participants (50%) completed all time points, and the remaining results were reported for this sample. The only significant demographic factors differences between study completers and non‐completers were for healthcare degree type (*χ* ^2^ [11] = 20.1, *p* = 0.045), with disability practice (*χ* ^2^ [1] = 7.1, *p* = 0.008) and public health unit students (*χ* ^2^ [one = 8.3, *p* = 0.004) less likely to complete the study.

### Research question 1: AASK outcomes (Potential effectiveness)

**FIGURE 2:**
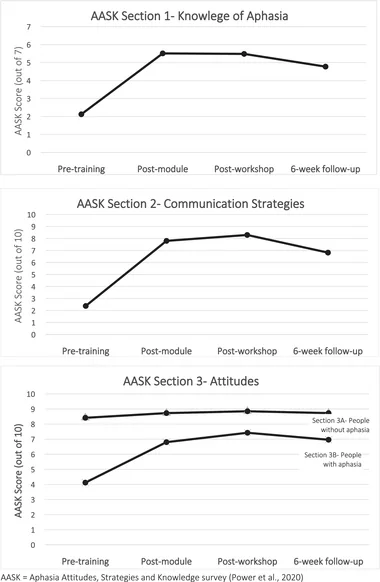
AASK scores over time for each section. Note: *Y*‐axis scales represent minimum and maximum scores for each AASK section. Error bars represent the standard error of the mean.

The first aim of this study was to investigate the potential effectiveness of the two‐part CPT by examining the variables measured by the AASK survey including aphasia knowledge, knowledge of communication strategies, and attitudes (confidence). Tables 2 and 3 present an overview of the AASK results relating to question 1, and Figure 2 illustrates the AASK score data visually at each time point.

**TABLE 2:** Aphasia Attitudes, Strategies and Knowledge (AASK) survey outcomes and results from post‐hoc paired *t*‐tests (*n* = 106).

**AASK survey**	**Time point (number)**	**Mean (SD)**	**Pre‐T (T1) to post‐M (T2) change**	**Pre‐T (T1) to post‐W (T3) change**	**Pre‐T (T1) to FU (T4) change**	**Post‐M (T2) to post‐W (T3) change**	**Post‐M (T2) to FU (T4) change**	**Post‐W (T3) to FU (T4) change**
Section 1: Knowledge of aphasia (/7)	Pre‐training (T1)	2.123 (1.224)	*t* = −22.369 *p* = < 0.001*** *d* = 2.158	*t* = −18.769 *p* = < 0.001*** *d* = 2.058	*t* = −15.583 *p* = < 0.001*** *d* = 1.602	*t* = 0.233 *p* = 0.817 *d* = 0.016	*t* = 5.134 *p* = < 0.001*** *d* = 0.413	*t* = 5.388 *p* = < 0.001*** *d* = 0.375
	Post‐M (T2)	5.509 (1.389)						
	Post‐WS (T3)	5.481 (1.526)						
	FU (T4)	4.774 (1.575)						
Section 2: Facilitative strategies (/10)	Pre‐training (T1)	2.377 (1.954)	*t* = −22.730 *p* = < 0.001*** *d* = 2.313	*t* = −24.472 *p* = < 0.001*** *d* = 2.582	*t* = −18.245 *p* = < 0.001*** *d* = 1.184	*t* = −2.918 *p* = 0.004** *d* = 0.224	*t* = 5.125 *p* = < 0.001*** *d* = 0.417	*t* = 7.398 *p* = < 0.001*** *d* = 0.656
	Post‐M (T2)	7.802 (1.833)						
	Post‐WS (T3)	8.292 (1.690)						
	FU (T4)	6.821 (2.088)						
3A: Attitudes (people without aphasia) (/10)	Pre‐training (T1)	8.415 (1.372)	*t* = −2.499 *p* = 0.014** *d* = 1.194	*t* = −3.451 *p* = < 0.001*** *d* = 0.274	*t* = −2.231 *p* = 0.028* *d* = 0.194	*t* = −1.841 *p* = 0.068 *d* = 0.086	*t* = 0.000 *p* = 1.000 *d* = 0.000	*t* = 1.120 *p* = 0.265 *d* = 0.089
	Post‐M (T2)	8.726 (1.183)						
	Post‐WS (T3)	8.849 (1.111)						
	FU (T4)	8.726 (1.167)						
Section 3B: Attitudes (people with aphasia) (/10)	Pre‐training (T1)	4.123 (1.750)	*t* = −16.290 *p* = < 0.001*** *d* = 1.356	*t* = −20.100 *p* = < 0.001*** *d* = 1.691	*t* = −16.792 *p* = < 0.001*** *d* = 1.411	*t* = −6.353 *p* = < 0.001*** *d* = 0.399	*t* = −1.267 *p* = 0.208 *d* = 0.098	*t* = 3.759 *p* = 0.001*** *d* = 0.293
	Post‐M (T2)	6.802 (1.298)						
	Post‐WS (T3)	7.425 (1.226)						
	FU (T4)	6.962 (1.407)						

*Notes*: AASK = Aphasia Attitudes, Strategies and Knowledge survey (Power et al., 2020); Pre‐T = pre‐training (AASK Time 1); Post‐M = post‐module—training Part 1 (AASK Time 2) Post‐W = post‐workshop—training Part 2 (AASK Time 3); FU = 6‐week follow‐up (AASK Time 4).

*p < 0.05, **p < 0.01, ***p < 0.001. Paired *t*‐test degrees of freedom = 105.

**TABLE 3:** Aphasia Attitudes, Strategies and Knowledge (AASK) survey outcomes and results from repeated measures MANOVA tests (*n* = 106).

**AASK survey**	**Wilk's lambda**	***F* (d.f.)**	***p***	**Partial eta squared**
Within‐subjects				
Time	0.083	86.818 (12, 94)	< 0.001^***^	0.917
Univariate tests				
Section 1: Knowledge of aphasia	–	225.817 (3)	< 0.001^***^	0.683
Section 2: Facilitative strategies	–	313.525 (3)	< 0.001^***^	0.749
Section 3A: Attitudes (people without aphasia)	–	5.165 (3)	0.002^**^	0.047
Section 3B: Attitudes (people with aphasia)	–	216.980 (3)	< 0.001^***^	0.674

*Notes*: AASK = Aphasia Attitudes, Strategies and Knowledge survey (Power et al., 2020).

* *p* < 0.05,

** *p* < 0.01,

*** *p* < 0.001.

Statistically significant improvement on all sections of the AASK (see Table C1 in Appendix C) were demonstrated between Time 1 (before training) and Time 4 (6‐week follow‐up). The outcomes for each AASK section at each time point (Table 3) are as follows. Following the online training module (part 1), there was a mean score increase of 3.4/7 for the AASK Section 1 which indicated sudents had improved their knowledge of aphasia including their ability, to identify four key features of aphasia, and were to describe how aphasia can impact on access to healthcare. In Section 2, participants were asked about their knowledge of facilitative strategies including their ability to identify three strategies to acknowledge competence, and seven strategies to reveal competence when communicating with people with aphasia. Following the online training module (Part 1), there was a mean score increase of 5.2/10 for AASK Section 2, meaning that on average participants were able to accurately list five more strategies. Attitudes to communicating with people *without* aphasia (AASK Section 3A, out of 10 marks) improved by 0.3, while attitudes exemplified by self‐reported comfort and confidence towards communicating with people with aphasia (AASK Section 3B, also out of 10) increased by a mean of 2.7. These changes reflected statistically significant improvement, with large effect sizes identified (apart from a small‐to‐medium effect for Section 3A) (Table 3).

After the workshop (Time 3), participants maintained gains from the online module in Sections 1 and 3A and made further statistically significant increases in Sections 2 (knowledge of facilitative strategies) and 3B (comfort and confidence interacting with a person with aphasia) (Table 2). At the 6‐week follow‐up survey (Time 4), all prior gains except Section 3A showed a statistically significant decline compared with post‐training scores (Table 2).

At the 6‐week follow‐up, participants were asked about their access to further CPT, and whether they had reviewed their CPT module. A total of 55 (51.9%) reported accessing at least one type of CPT or resource between completing the module and post‐module survey (Time 2), while *n* = 49 (46.2%) viewed materials between the post‐module and post‐workshop survey (Time 3) and *n* = 33 (31.1%) between the post‐workshop survey and follow‐up survey (Time 4) (see Table C1 in Appendix C).

In summary, there were significant gains after the module, with maintenance/modest further gains after the workshop. At the 6‐week follow‐up, there was a significant decline compared with post‐training levels, but outcomes were still significantly improved compared with before training.

### Research question 2: Perceptions of CPT: feedback survey (Acceptability)

The second research question explored participants’ perceptions of the training, ascertained from a feedback survey administered after the workshop (see Table D1 in Appendix D and Figure E1 in Appendix E). Of 236 participants, 161 (68%) completed the survey. The 13 Likert‐scale items were rated as either *agree* or *strongly agree* by over 80% of respondents (one item: 100% of respondents, 12 items range: 80–98% of respondents) (see Figure E1 in Appendix E). Ratings for *disagree* or *strongly disagree* occurred for seven items (six items range: 1–3%; 1 item 9%). Summarizing the open‐ended response section indicated students valued the relevance of the training, learning about aphasia and strategies to apply, and having videos and discussion to engage with. In some cases, marked shifts in understanding were evident from the training including one student's comment:

Strong guidance and teaching in understanding that people with aphasia are competent and intelligent. I think before this I would have automatically assumed that healthcare decisions need to be made with other family members or friends, but I now have a different mindset around what these people can do.

Regarding suggested improvements, participants wished to learn more about aphasia and other communication impairments and apply newly learnt knowledge in the form of more detailed skills practice with a clinically relevant focus.

## DISCUSSION

### Outcomes of completing the two‐part CPT (Potential effectiveness)

A two‐part online aphasia CPT package (up to 110 minutes) for a multidisciplinary sample of student healthcare professionals was potentially effective in improving (a) knowledge of aphasia, (b) knowledge of facilitative communication strategies for engaging with people with aphasia, and (c) attitudes towards communicating with individuals with and without aphasia as measured by the AASK survey (Power et al., 2021). This finding supports the hypothesis that completing Part 1 of the training (the 50‐minute asynchronous self‐directed online module) would lead to significant improvements across concepts measured in the AASK (Time 2). Our hypothesis that Part 2 of the training (the 1‐hour online facilitated workshop) would result in further improvement (Time 3) was partially supported. Improvements were demonstrated for AASK Section 2 (knowledge of facilitative communication strategies). While post‐module gains for Sections 1 (aphasia knowledge) and 3 (attitudes) were maintained, no significant additional improvements were demonstrated on Sections 1 and 3.

A unique part of this study not included in other research to date was the measuring of outcomes at an extended follow‐up period. The hypothesis that post‐training improvements would be maintained 6 weeks later (Time 4) was not completely supported, though results at the 6‐week follow‐up phase still remained significantly better when compared with pre‐training scores (Time 1). The improved scores from baseline to follow‐up, in this study, demonstrate longer term, educationally meaningful change within a large sample across a wide variety of healthcare professional disciplines. This builds on previous work (Power et al., 2021) and is consistent with the findings that training delivered online through self‐directed learning can support effective increases in knowledge (Decorby‐Watson et al., 2018).

The online aphasia‐specific lecture of 50–60 minute duration was sufficient to lead to knowledge, confidence, and attitudinal change, in support of previous studies that have used similar brief lectures in both face to face or online modalities (Cameron et al., 2015; Finch et al., 2020; Power et al., 2020). This finding contrasts with other research in CPT training for broader range of communication impairments that found lectures alone did not lead to an increased ability to list facilitative strategies (e.g., Forsgren et al., 2017; Saldert et al., 2016). In the present study, the 50‐minute workshop added modest benefit (mean 0.5‐point increase out of a possible total 10 marks) regarding students’ knowledge of facilitative strategies. This was potentially due to its focus on additional revision and opportunities to observe, evaluate, and discuss video interactions. Two areas were maintained post‐workshop without showing further gains (i.e., Section 1—Knowledge of aphasia and Sections 3A and 3B—Attitudes towards communicating with people without and with aphasia). Possible explanations are that aphasia knowledge was sufficiently addressed to provide knowledge‐based learning. However, further improvement regarding attitudes (confidence) may require more extensive, skills‐based training including conversation practice and feedback to provide sufficient experiential learning to result in a feeling of increased confidence in those skills. The decline in scores at follow‐up suggests that revision is required to maintain learning, consistent with other research, even when skill acquisition is a direct focus of training (e.g., Offiah et al., 2019). These points support the argument that student healthcare professionals can benefit from CPT incorporated throughout their degrees, embedded within the curriculum in relevant subjects or as prerequisite training before relevant clinical placements. However, based on our results, students may require refresher training sessions if they had their CPT more than 6 weeks before a placement.

In terms of research question 2, feedback survey results reflected good to high acceptability of the program overall, with multiple responses highlighting the usefulness of being able to engage with a topic area predominantly absent from current coursework. The present study achieved a substantial degree of participant uptake, with nearly one‐third (266) of the potential 845 students invited to participate completing the first survey. There was moderate to low retention to the end of training (*n* = 144) and up to the follow‐up point (*n* = 106). This finding contrasts with other studies that have reported low student participant uptake but good retention (e.g., Finch et al., 2020), although these studies often only required retention for 2 shorter assessment time points. The present study's participant retention rate was deemed particularly positive in the context of the significant challenges students experienced remaining engaged with course work during the highly disruptive COVID‐19 pandemic (Aboagye et al., 2021). While we did not conduct formal feasibility analysis, there was strong uptake of the learning materials by academic teaching staff. Eight of the 12 disciplines invited to use the learning materials chose to embed the coursework as part of their curriculum. The remaining four disciplines provided the training as an optional learning experience for students. In respect to perceived value by students, two thirds of student participants were drawn from courses for whom the CPT training and research was an optional extra. This suggests that students themselves identified value and relevance to their current and future health‐related work activities.

### Limitations and future directions

Most participants in the sample who completed the AASK at all time points (*n* = 75, 70%), self‐selected for the training (as opposed to it being embedded within their coursework). The occupational therapy and physiotherapy student participants formed the largest proportion of this group and the greatest number of students overall. Therefore, the current sample could overrepresent individuals who may be more interested in the topic and are enrolled in two allied health degrees who very commonly work with stroke survivors with aphasia. There was attrition from all degrees between 33.3% and 82.4% and no clear pattern of attrition could be ascribed to specific degree cohorts. Specific data were not collected regarding participant numbers for the workshop, which impacts the capacity to understand retention rates in more detail. Further, though participants were routinely instructed not to refer to learning materials while completing the AASK surveys, this could not be monitored remotely. Given that results were not maintained between post‐workshop and follow‐up phases (Times 3 and 4), this may suggest that participants had not re‐engaged with materials during the specified period and that consolidating skills‐based experiences may be required.

While it is encouraging that the workshop led to slightly improved knowledge of facilitative strategies beyond that acquired on the module, the absence of a randomized controlled trial design and not controlling for information seeking and/or revision practices makes it difficult to discern the relative impact of the two training components. Further, it is likely that extending the length and adapting the workshop to incorporate a conversation practice component with people with aphasia (e.g., Cameron et al., 2015; Finch et al., 2020; Harmon et al., 2015; Welsh & Szabo, 2011) would support development of facilitative skills and improved attitudes to communicating with people with aphasia.

Such skills practice with a person with aphasia may be a key component of CPT (see Isaksen & Randrup‐Jensen, 2018) not addressed here. The present study consisted of active learning role‐play exercises in the workshops to address skills acquisition to some degree. However, it was not feasible to arrange individual conversations with people with aphasia to apply the online and workshop learning, nor to establish skills outcomes evaluation with this large number of participants (*n* = 236 at initial baseline assessment time point). In addition to the skills practice intervention, the assessment of skills practice would require the participation of people with aphasia across four assessment time points. Conservatively, involvement of people with aphasia in the intervention and assessment time points would require 300 hours of direct time, notwithstanding the associated training, debriefing and additional administration hours. This issue is a critical scale‐up implementation challenge for both the training and evaluation of skills in large cohorts. The use of standardized simulated patients may mitigate this to a degree; however, this would still require substantive resourcing. Future investment in technological solutions such as avatars (e.g., https://dementialearning.org.au/technology/talk‐with‐ted/support/) may offer a potential solution to this scale‐up challenge—however, this area needs further research. While skills practice is important in achieving specific CPT outcomes, there are, overall, multiple desirable CPT outcomes. The reporting of attitudes and student quotes identified above, in relation to an increased understanding of inherent competency of people with aphasia and stated future actions that would engage them directly with people with aphasia, are important outcomes of CPT. Further research may examine the degree to which an enhanced ability to acknowledge inherent competence does or does not lead to an increased willingness to engage with people with aphasia during healthcare interactions.

The question of the relative contribution of the more lecture‐based content and active practice on outcomes is still not clear in the student CPT literature. Our research suggests the substantive improvement in outcomes was delivered by the online learning, which did also incorporate videos and reflective elements at a similar dose (50 minutes) to the workshop. A previous study (Finch et al., 2018) demonstrated that students who received a knowledge‐based lecture (60 minutes), combined with a 10–15 minute discussion with a person with aphasia achieved significantly better confidence and knowledge outcomes than the students who completed the discussion with the person with aphasia alone. The knowledge component appeared to be a critical foundation to support more skills‐focused subsequent training elements. However, in that study, students randomized to the conversation only group had substantially less time overall spent engaging in CPT learning and so the outcome may have merely been a result of difference in ‘dose’. Future research is needed to establish the critical elements of active learning tasks that would add value and increased outcomes within the context of student CPT.

Finally, while we have included a wide variety of healthcare professions, future research should focus on training student healthcare professionals from a still wider range of professional disciplines (including nursing, medicine, and social work), ascertaining efficient training delivery mechanisms (lecture, workshop, simulated practice) to realise desired outcomes, and investigating impacts of CPT for students undertaking clinical placements.

## CONCLUSIONS

The results of this study show that a brief online aphasia CPT package has the potential to improve student healthcare professionals’ preparedness to deliver healthcare to people with aphasia through improved knowledge of aphasia and facilitative strategies as well as attitudes towards communicating with people with aphasia. It is recommended that CPT be incorporated into university curricula before clinical placement opportunities for all student healthcare professionals, and embedded over time to ensure maintenance of outcomes that are integral to positive healthcare interactions for people with aphasia.

## CONFLICT OF INTEREST STATEMENT

The authors declare they have no conflict of interest.

## Supporting information

SUPPORTING INFORMATION

## Data Availability

The data that support the findings of this study are available from the corresponding author upon reasonable request.

## APPENDIX A

Aphasia Attitudes, Strategies and Knowledge (AASK) survey, originally published as Power, E., Falkenberg, K., Elbourn, E., Attard, M., & Togher, L. (2021) The test–retest reliability of the Aphasia Attitudes, Strategies and Knowledge (AASK) survey with student health professionals. *Aphasiology*, 35(9), 1190–1206. Reprinted with kind permission. See original answer guide below and Supplementary file for expanded scoring guidance.

## APPENDIX B

**TABLE B1:** Description of the communication partner training package using the Template for Intervention Description and Replication (TIDieR) (Hoffmann et al., 2014)

1. Brief name	Communicating with People with Aphasia in Healthcare Contexts (adapted from Supporting Conversation for Adults with Aphasia [SCA™] (Kagan, 1998)
2. Why	Receiving training in supporting individuals with aphasia to communicate more effectively (and to communicate more effectively with them) can lead to more successful transfer of information and enhanced social connection. Training student healthcare professionals in this area supports learning beyond what may be addressed in the context of their degree
	The program, Communicating with People with Aphasia in Healthcare Contexts, comprises a 50‐min online module and a 1‐h online interactive workshop. It was hypothesized that both components of the training would be of value over one alone
	SCA™ (Kagan, 1998) recognizes the right to access conversation and its inherent importance to daily life. The training is based on the notion of ‘conversational partnerships’, with shared roles and success as interdependent rather than individual—resulting from a combination of skill and experience (of the partner with aphasia, and the partner without aphasia) and resource availability. Training therefore addresses, in particular, generic skills the partner without aphasia can develop and apply with a range of aphasia presentations, and augmentative and alternative resources to support communication. The ultimate goal of SCA™ is increased communicative confidence and participation for the person with aphasia
	For additional theoretical links associated with SCA™, see Kagan (1998) and Cruice and Kate (2019)
	The module is predominantly didactic but includes reflective questioning and passive skill‐building to support learning (O'Rourke et al., 2018). The workshop builds on this approach with the added opportunity to consolidate information and engage in discussion with peers and the speech–language pathologist
	The training program Communicating with People with Aphasia in Healthcare Contexts differs from the comprehensive SCA™ training in that active skills practice and feedback on conversation practise is not currently included
	From the perspective of experiential learning theory, the present training likely address three of Kolb and Fry's (1975) four ‘learning environments: affectively‐oriented (feeling), symbolically‐oriented (thinking), and perceptually‐oriented (watching)’. The two tasks theorized to occur in these environments are ‘rasping’ (involving concrete experiences and abstract conceptualization) and ‘transforming’ (involving reflection and action). Given the absence of skills practice, the fourth environment—behaviourally oriented (doing) and associated tasks—are not likely to be actively realized
3. What (materials)	Module: Online ‘Moodle Book’ based on the training reported by Power et al. (2020). Written information was presented using multiple modalities including text and a video demonstration. Additionally, text and video commentary were included to highlight specific communication strategies or processes and provide opportunity for reflection
	Workshop: The workshop was delivered using Microsoft PowerPoint. It included a combination of information provision, video observation of additional aphasia presentations, note‐taking (as a whole group and in a ‘breakout room’) and discussion
	Further information regarding the training package can be obtained from contacting the last author
4. What (procedures)	Module: Participants completed the module by navigating through the Moodle Book. Participants were encouraged to reflect on questions posed and take notes throughout the module. Participants were allowed to actively review their notes and/or seek information regarding aphasia between data collection periods, including between the module and the workshop sessions
	Workshop: The workshop alternated across didactic slides presented by the speech–language pathologist, video‐watching and opportunities for discussion. Participants were encouraged to take notes, share ideas/comments and ask questions throughout the workshop
5. Who provided	The online workshop was delivered by a qualified speech–language pathologist (first author) who has experience working with adults with acquired neurogenic communication disorders
6. How (mode of delivery; individual or group)	Module: Accessed independently via the participants’ university learning management system. They were asked to complete this in their own time
	Workshop: Delivered via Zoom. Participants either participated in the workshops as part of usual class groups during class time (where completing the training as an embedded part of their course curriculum) or a mix of usual class groups and/or mixed cohort groups (where completing the training as an optional, extracurricular activity) Across the 13 workshops conducted, group sizes ranged from approximately 10 to 50 participants
7. Where	Module: Participants could complete this at a location of their choosing
	Workshop: Sessions were conducted over Zoom during a remote learning period due to COVID‐19, with the participants located at individual private spaces (likely their homes), and the clinician in a quiet, private space
8. When and how much	Module: Estimated to take 50 min to complete. Completed before attending the workshop
	Workshop: 1 h in duration. Total of 1 h 50 min (not including any additional revision or information‐seeking time undertaken by participants outside of the training)
9. Tailoring	As the training workshop were presented live to a diverse set of participants across 13 sessions, facilitator flexibility was allowed in relation to the degree of time spent on each section and the order of content provided within the 1‐h timeframe. Time spent on each section was dependent to a degree on the number and nature of questions asked and discussion occurring
10. Modification	A pilot version of the training workshop (version 1) was provided to 16 postgraduate dietetics students, following which adjustments were made to the workshop content to manage time constraints. The present manuscript details a separate set of participants and results pertaining to version 2. No adaptations or tailoring of content occurred to the version 2 workshops
11. How well (planned)	Participant retention: Measured by the number of participants who completed each of the assessment phases—including those after the two training components, shown in bold (pre‐AASK, post‐module AASK, post‐workshop AASK, 6‐week follow‐up AASK) It was not possible to record the number of participants who completed the module, and a formal roll was not taken at the workshop
	Treatment (training) fidelity: Steps were taken to ensure fidelity across the training workshops. Video recordings were captured and chat function text was saved, and an independent rater completed a fidelity checklist while viewing 20% (3/13) of the workshops (re‐labelled to blind to workshop number) The checklist addressed the presence or absence of nine content elements (e.g., *Learning aims are verbalised*) and seven process elements (e.g., *Students provide observations and/or reflections regarding video exposure to person with aphasia*) Of these 16 elements, 12 were deemed ‘essential’ and four ‘desirable’. Further to this, the rater noted the length of time spent addressing each element and added annotations
12. How well (actual)	Participant retention: The original sample consisted of 271 participants (completing the Demographics survey) At the post‐module AASK phase, a retention rate (of the original sample completing the pre‐AASK) was 76.7%. At the post‐workshop AASK phase, the retention rate was 61%, and it was 38.6% at the 6‐week follow‐up AASK phase
	Treatment (training) fidelity: The three training workshop recordings randomly selected for fidelity checking were 4, 12 and 13 of 13. Across each of these, all six ‘essential’ *content* elements (e.g., *Learning aims are verbalised*) and all six ‘essential’ *process* elements (e.g., *Students provide observations and/or reflections regarding video exposure to person with aphasia*) were noted to be present. For the ‘desirable’ elements, two of the three *content* elements and the one *process* element were present across the three workshops (one content element was absent in workshops 12 and 13) Annotations indicated that presenter time spent on various sections differed somewhat between workshop 3 compared with 12 and 13, with the latter involving less time on recapping concepts, and more time on reviewing and discussing the video recordings and discussing clinical application of supported communication

**APPENDIX B REFERENCES**

Hoffmann, T. C., Glasziou, P. P., Boutron, I., Milne, R., Perera, R., Moher, D., et al. (2014) Better reporting of interventions: template for intervention description and replication (TIDieR) checklist and guide. *BMJ*, *348*(mar07 3), g1687–g1687. http://doi.org/10.1136/bmj.g1687

Kagan, A. (1998) Supported conversation for adults with aphasia: Methods and resources for training conversation partners. *Aphasiology*, *12*(9), 816−830. http://doi.org/10.1080/02687039808249575

Kolb D.A., Fry, R. (1975) Toward an applied theory of experiential learning. In C. Cooper (ed) *Theories of Group Process*. John Wiley.

Power, E., Falkenberg, K., Barnes, S., Elbourn, E., Attard, M. C., & Togher, L. (2020) A pilot randomized controlled trial comparing online versus face‐to‐face delivery of an aphasia communication partner training program for student healthcare professionals. *International Journal of Language and Communication Disorders*, *55*(6), 852−866.

## APPENDIX C

**TABLE C1:** Summary of participants’ reported review of communication partner training materials and seeking additional content over time (*n* = 106).

	**Reported review of materials**		**Nature the of how participants reviewed materials or sought further content for ‘Yes’ (*n*)**				
**Time point**	**‘No’, *n*, % of total**	**‘Yes’, *n*, % of total**	**(a) Reread/skimmed notes made from module and/or workshop (depending on time point)**	**(b) Explored one or more physical reference materials** **^a^**	**(c) Other** **^b^**	**Combination of (a), (b) and/or (c)**	**Of ‘yes’, nature not indicated**
Between module and **post‐module AASK (Time 2)**	51, 48.1%	55, 51.9%	32	4	1	16	2
Between post‐module AASK and **post‐workshop AASK (Time 3)**	57, 53.8%	49, 46.2%	35	3	0	11	0
Between post‐workshop AASK and **6‐week follow‐up AASK (Time 4)** ^c^	72, 67.9%	33, 31.1%	25	3	0	6	0

*Notes*:

^a^ Reference materials, e.g., information or resources on internet websites; coursework; textbook.

^b^ Other, e.g., discussion with/explanation of content to others, made a summary of earlier notes.

^c^ There were missing data for *n* = 1 at this time point (did not respond regarding review)—% based on *n* = 105 respondents rather than *n* = 106.

AASK = Aphasia Attitudes, Strategies and Knowledge survey (Power et al., 2020); bold indicates survey completion time point of interest.

## APPENDIX D

**TABLE D1:** Feedback survey open‐ended responses: Example comments regarding what was most valuable or meaningful about the communication partner training package.

Responses to the items, ‘*What did you find most valuable or meaningful about the training package?’* and ‘*Please feel free to expand on any of the points addressed via the rating scale’*	
**Broad content category**	**Examples**
Relevance/application, value	‘Doing the module before placement would have been really useful as I saw some patients with aphasia and did struggle with communication.’ ‘I really valued learning about how to communicate with people who have communication disabilities as I feel that this will be very helpful in my work and placements in the future.’ ‘… I could easily transfer some of the techniques taught to other communication difficulties also.’ ‘Being able to apply the info to previous experiences I've had with patients with aphasia and being able to reflect on that and come up with ideas on how I can improve similar interactions in the future.’
Conceptualizing aphasia and people with aphasia	‘Strong guidance and teaching in understanding that people with aphasia are competent and intelligent. I think before this I would have automatically assumed that healthcare decisions need to be made with other family members or friends, but I now have a different mindset around what these people can do.’ ‘Just simply being informed about Aphasia and how to communicate with someone living with it (because I was unaware of the condition prior).’ ‘It … showed how different aphasia [sic] can present in different patients, which was useful to see.’
Communication strategy knowledge	‘It just helped me learn about some effective techniques to use for aphasic clients. Before the training, I wouldn't have known where to start.’ ‘I thought it was good to explicitly consider some of the issues and strategies one may use. I feel like most of the strategies were fairly simple and should be incorporated with a range of clients, not just those with aphasia.’ ‘This is the kind of issue that comes up every now and again for Psychologists and as such it's important to know about it and to work out how to handle aphasia and other communication difficulties in advance because you might not have advanced warning or time to research relevant strategies.’
Recorded content	‘In lieu of physically having consumers who have aphasia support us in our learning, having videos of consumers interacting with health practitioners and family members was very helpful, as this helped to put the theory into practice.’ ‘The video's [sic] demonstrated that this can be a really meaningful way to improve the quality of care for a person with communication difficulties.’ ‘Most of my understanding came from the recorded element, however having the opportunity to view and break down ways to assist with the communication in the workshop element allowed me to practice what I had learned.’
Content style	‘I liked how everything was broken into small segments as it made it seem more doable and made it easier to work through (as opposed to if everything was one video) Seeing that a video was only 1 min made it easier to finish everything in one sitting.’ ‘… key ideas were highlighted which allowed me to rectify if I had taken away the most valuable points.’
Module‐specific comments	‘I found it very valuable that we were provided with written information and then it was reinforced with a video example right after, it really consolidated understanding.’
Workshop‐specific comments	‘The ability to ask any questions surrounding the module content to the workshop trainer. This helped solidify my understanding of the content.’ ‘I found splitting into break out rooms via Zoom to discuss the patient was the most meaningful as we were able to discuss and apply the theory into practice.’
Trainer characteristics/approach	‘It was motivating and reassuring to have the facilitator present as very friendly, open and informative. I felt very comfortable in the workshop.’ ‘The teaching in the workshop was engaging and you did very well to minimize the awkwardness of a zoom [sic] class with strangers.’
Responses to the items, ‘*Would you recommend any changes to the training package? (What would make it better?)’* and ‘*Please feel free to expand on any of the points addressed via the rating scale’*	
**Broad content category**	**Examples**
Module content	‘As an educated English‐speaking person, the recorded element of the package felt quite slow and repetitious. However, knowing that not all professionals who may access the package have English as their first language, it was pitched well to accommodate this.’ ‘More videos and quiz with scenarios at the end of the module.’
Module style	‘Different coloured and sized text was confusing at times when opened in [name of university Learning Management System]’
Workshop content and length	‘… the workshop I found a little repetitive as to the same content in the module…’ ‘… some sort of lived experience or narrative of a real person living with aphasia might stimulate greater engagement/interest and further stress the humanity at play.’ ‘Having an opportunity to practice these strategies with people with aphasia …’ ‘More activities where we get to try the strategies and role play as both client and clinician would be valuable in learning these skills and strategies. It might also be worth making time after people do these kinds of activities to ask questions for the purposes of troubleshooting issues that people come up against.’ ‘It would be really great to see some real‐life scenarios in a ward or during a session—although the ones provided were still great and useful, it would be beneficial to see a professional navigate the situation with all that goes on around them in different situations that aren't so controlled.’ ‘Perhaps include more video examples from a variety of contexts (e.g., children, special needs, etc.).’ ‘… the workshop needs more time, especially to include activities and role plays. It might also be worth doing 2 workshops if one 2hr workshop isn't feasible.’ ‘A longer workshop to allow for more questions to be raised and for content to be discussed in relation to the profession/area of study i.e., psychology, dietetics.’
Workshop delivery	‘The opportunity to discuss different examples of people with aphasia further in the small breakout room format.’ ‘The zoom [sic] workshop was a bit difficult to participate in through no fault of anyone. Zoom can sometimes seem a little impersonal so I would recommend having the workshop face to face in future (if the state of the world allows it) to allow for free‐flowing conversation of ideas^a^.’

*Note*: Four students indicated in the open‐ended responses that a face‐to‐face workshop would be preferred to online delivery.
