# Supplementary material for: Efficacy of online communication partner training package for student healthcare professionals
Source: Int J Lang Commun Disord. 2023 Sep 3;59(1):304–26. doi: 10.1111/1460-6984.12947 (PMC10952497; doi:10.1111/1460-6984.12947)
Supplement: Supplementary file 1 — SUPPORTING INFORMATION [file JLCD-59-304-s001.pdf]

Aphasia Attitudes, Strategies and Knowledge (AASK) - Expanded scoring guidance - 2023

This document is designed to expand upon the content already provided in the original publication by Power, E., Falkenberg, K., Elbourn, E., Attard, M. C., & Togher, L. (2020). The test–retest reliability of the Aphasia Attitudes, Strategies and Knowledge (AASK) survey with student health professionals. *Aphasiology, 35*(9), 1190-1206, DOI: 10.1080/02687038.2020.1787943

The information in the original paper is not repeated here directly due to copyright restrictions. The information below should be used in conjunction with the original answer guide in the above article.

Please contact Emma Power at emma.power@uts.edu.au for further information about AASK.

Note: PWA = Person with aphasia

<table>
<tr><th>SECTION</th><th>GUIDANCE</th></tr>
<tr><td>GENERAL</td><td>

Responses needed to be relevant to the section being completed (e.g., strategies relating to revealing competence (supporting the PWA's understanding and/or expression) were not awarded points if they were listed under the section relating to acknowledging competence (showing respect and support).
Where responses included a mix of incorrect and correct content, the item was scored a 1 for the correct content. It is recommended that the section receiving credit is marked in some way for clarity. For example, for the following response to Question 1.1 (Identify four key features of aphasia): "People with aphasia have suffered a stroke or a damage to their language part of their brain which sometimes affects their motor skills such as face movements." (content in bold is what constitutes score of 1)
To enhance intra-rater reliability, we encourage raters to score by item rather than by participant and look over last 10 or so responses for a single item to confirm consistency as they go.

</td></tr>
<tr><td>Q1.1.
Identify four features of aphasia</td><td>
Answer guide (possible total of 4 marks) can include

Aphasia is a <u>language problem</u>
Aphasia affects a person's <u>ability to communicate</u> with others
People with aphasia can have <u>difficulties with</u> understanding, speaking, reading, and/or writing

Reference to 'speech' in context of pronunciation/articulation/sounds is incorrect, though 'affect ability to speak', 'speaking', 'talking', 'saying' (where it's clear that 'communication' is the concept) is acceptable

E.g., 'Difficulty speaking with people' = 1, 'can't verbally say the word(s) they want' = 1.
Some responses may be too specific or too vague e.g., "words don't come out as intended"—too specific re. difficulty speaking; "word finding difficulty—too specific re. difficulty speaking"

if these sorts of deficit areas are listed as <u>separate</u> points/key features, only allocate 1 mark (as this is only one of the possible response types for the question)

1 mark can be awarded even if the deficit set (difficulties with understanding, speaking reading and/or writing) is incomplete (however, something related to expression AND understanding <u>must</u> be included as a minimum to reflect acknowledgement of multi-component/modal deficit)

E.g., "problems reading and writing" = 1 because it includes receptive and expressive components

terms/concepts listed in isolation without notion of deficit/difficulty e.g., "processing language", "language", "reading" = 0.

It is caused by damage to the areas of the brain involved with processing language

Some respondents may refer to causes including stroke, TBI, tumour; If a cause is listed without reference to injury/damage/impairment/disability, can still score as 1 if it is plausible

E.g., "brain damage", "stroke" = 1

</td></tr>
</table>

	○ *If respondents refer to other general features of aphasia such as:*<br>▪ *the fact it is **acquired***<br>▪ *that it is **different** for everyone*<br>▪ ***co-morbidities** such as hemiparesis/motor speech deficits.*<br><br>*These are not considered '**key** features of aphasia' itself (for instance, many disorders are acquired—the notion that it involves brain damage is more specific to aphasia) so in these cases score 0.*<br><br>• ***Aphasia in itself does not impact intellect***<br>○ (Needs to be specific!) e.g., "does not affect the general ability of the person" is too vague = 0<br>○ Reference to 'cognition' is acceptable e.g., "cognition is maintained" = 1<br><br>***Example response set + scoring***<br>• "Difficulty with using expressive and/or receptive language *(1 mark as the minimum concepts are addressed)*, and word finding difficulty *(0 marks as overlaps with expressive language)*, without loss of intellectual function *(1 mark)*."<br><br>*Note: These marks are allocated even though there are multiple separate concepts in one response.*<br><br>*(Total 2 of 4 marks awarded)*
**Q 2.1**<br>**List THREE strategies that you can use to ACKNOWLEDGE the competence of a person with aphasia**	**Answer guide can include:**<br><br>• ***Speak naturally***<br>○ *'Don'ts' relating to respectful communication are also permissible (e.g., don't talk down to them / don't treat them as though they are less intelligent)*<br>○ *Other behaviours that suggest the person is a competent adult are also acceptable, e.g.:*<br>▪ Treat the person like an adult<br>▪ Treat them like you would any other client<br><br>• ***Acknowledge the frustrations of the person with aphasia***<br>○ Related concepts like "acknowledge the person's aphasia"/ "acknowledge it can be hard" are also acceptable<br><br>• *Convey you are dedicated/committed to the interaction (convey you are both in the conversation together as a team)*<br>○ Related examples:<br>▪ "Let them know you will 'stay the course' and work with them to achieve communication" = 1<br>▪ "Encourage them to keep at it when they are having difficulty" = 1<br>▪ E.g., that is less explicit (requires more interpretation): "tell them they can take their time" = 0<br><br>• ***Check that it is OK to seek information from others***<br>○ Respondents may also refer to "speaking to the person directly" which also reflects the concept of starting with the PWA first (not deferring automatically to another partner). These sorts of concepts = 1<br><br>• ***Acknowledge and be aware of your own limitations as a communicator***<br>○ Respondents may refer to concepts like "take responsibility for your part/role in the conversation"—this is also acceptable.<br><br>• ***Acknowledge the person with aphasia's intelligence / capacity to communicate/ capacity to make decisions***

- *An example of 0: 'acknowledge their understanding'—this is on the right track but too specific to comprehension, so misses the mark*

<u>Note</u>: general concepts that are included within the question itself e.g., "being respectful", "showing respect", "acknowledge their competence" = 0. The response needs to provide a 'how' this attitude will be achieved since the focus is on strategy use.

Example response set + scoring

- Acknowledge that they have the ability to communicate *(1 mark for response relating to "acknowledge their intelligence"/"I know you know")* but it is difficult for you to understand and for them to get their message across *(1 mark for response relating to "acknowledge their difficulty")*, and that this is a common problem that just requires strategies to overcome *(1 mark for response relating to "show you are dedicated to the interaction"). Total 3 marks contained in 1 response box.*
- Ask them how they would like to communicate whether it be using written language or drawings. *(1 mark as it could be considered relevant to acknowledging communication capacity and deferring choice to them)*
- Clarify any misunderstandings using other techniques *(0 as these better fit under 2.2 or 2.3; but note—if this has included concepts like 'respectfully', or 'acknowledging that you may not have understood' then can mark as 1, but as it stands this example is transactional)*

  (Total: 4 correct responses, 1 more than the 3 possible marks obtainable→3/3)

Q 2.2.

List THREE strategies that you can use to ensure a person with aphasia UNDERSTANDS what you are saying to them, i.e., strategies to get your information IN. If you are unsure, tick "Unsure".

***Answer guide –can include*:**

- ***Speak in short/simple sentences***
  - *Note: something like 'speak clearly' is not enough here.*
  - *"Slow down" etc would = 0. This is because this contradicts "speak naturally" addressed in 2.1.*
    - *HOWEVER,* "slow down" would be ok IF they noted something like "…IF the PWA appears to benefit from this/asks for slower pace to support their understanding".

- ***Use/add gestures, key written words, and/or pictures to your speech***
  - *Note: Explicit reference to adding TO ORAL OUTPUT is not required*
  - *If modes are listed as <u>separate</u> responses (e.g., 'Use gesture'; 'Add key words to your talking', mark only 1 (as this complete item [Add gestures, key written words, and/or pictures to your speech] is about the general reference to augmenting speech with other modalities).*

- ***Minimise potential distractions in the environment***

- ***Observe facial expression, gaze, and gesture closely for signs of understanding***
  - *Respondents may also refer to "looking for clues/cues" <u>regarding PWA's understanding,</u> or explicitly <u>checking</u> with them for their understanding*
  - *Responses that relate to "summarising" or "verifying" <u>to confirm the PWA's understanding</u> are also correct (i.e., could serve as a 1 for this response category—not as an <u>additional</u> mark)*
    - E.g., "ask them to repeat the key things you told them" = 1

- ***Give them time** to process your message*
  - *Be patient/don't rush*
  - *(Note: This is response acceptable in both 2.2—supporting understanding/message in, and 2.3—supporting expression/message out)*

- ***Ask if they would like/if you can seek out a family member/friend/other professional to help***
  - *Note: It is ideal if respondents refer to checking with the PWA first, but that is essentially a respect/competence element and the notion of seeking assistance to support information transfer is sufficient for 2.2 (support PWA's understanding/message IN) and 2.3 (support PWA's expression/message OUT)*

	Example response set + scoring<br>• Use small chunks of information *(1 mark)* and ask them if they understand *(1 mark)*<br>• Ask them to relay the information back to you in any way they can *(0—no need to add a mark as overlaps with second point in above response—though if the earlier one was not present, this item here would get 1 mark).*<br>• Use key words written down or pictures as other modalities to increase chance of understanding *(1 mark).*<br><br>*(Total 3 of 3 marks awarded)*
**Q 2.3.**<br><br>**List FOUR strategies that you can use to ensure a person with aphasia can adequately EXPRESS themselves, i.e., strategies to get their information OUT. If you are unsure, type "Unsure".**	**Answer guide – can include:**<br><br>• ***Ask questions in a logical order, from general to specific***<br>○ Respondents may also refer to this as "funnelling down from general to specific", or "asking questions using scaffolding"—either are acceptable if they can be linked back to this concept<br><br>• ***Ask "yes/no" questions***<br><br>• ***Ask fixed choice questions***<br>○ Note: As these are two different types of questions, 1 mark is awarded for each, even if they are listed in the same response line<br>○ Some respondents may refer to concepts like "asking strategic questions"—this is also acceptable IF they indicate what this might entail (e.g., "ask fixed choice questions"; "ask questions from general to specific", "ask questions using scaffolding")<br>○ Wording e.g.: "List options of answers for them to agree or disagree with" = 1<br><br>• ***Ask one thing at a time***<br><br>• ***Give the person with aphasia time to respond***<br><br>• ***Ask the person with aphasia to give you clues (e.g., with gestures, pictures, writing)***<br>○ *Regarding alternative wording: some respondents may refer to "giving PWA a way to communicate" / "ensuring there are materials available to support expression" / "use AAC supports" = 1*<br>▪ *Mention may occur of "acting like a detective" but this is only acceptable if it is linked to the notion of "looking for clues" or observing other modes of communication*<br>• E.g.: "If the person with aphasia gets stuck, think of yourself a bit like a detective and ask them to give you clues"<br>*(The key notion here is about augmented communication/a mode other than the current one being applied for the PWA's expression to be enhanced. A mark is granted even if the wording does not refer to the partner ensuring these means are available/providing these means—e.g., "pictures/visual aids")*<br>○ *If modes are listed as <u>separate</u> responses (e.g., 'suggest gesture'; 'writing', mark only 1 (as this complete item is about the general reference to augmenting speech with other modalities).*<br>○ *(Note: This response is acceptable in both 2.2—supporting understanding/message in, and 2.3—supporting expression/message out)*<br><br>• ***Summarise their message, and ask them to confirm it is correct***<br>○ *In this case the two points need to go together, because confirming <u>your</u> summary of <u>their message OUT</u> is about helping with the accuracy of their expression. If you are summarising without confirming accuracy of their output, then it's less about their expression and more about their understanding (2.2 supporting PWA's understanding/message IN).*<br><br>• ***Ask if they would like/if you can seek out a family member/friend/other professional to help***

	◦ *Note: It is ideal if they refer to checking with the PWA first, but that is essentially a respect/competence element and the notion of seeking assistance to support information transfer is sufficient for 2.2 (support PWA's understanding/message IN) and 2.3 (support PWA's expression/message OUT)*<br><br>• ***Minimise potential distractions in the environment***<br>◦ ***(****Note: This response is acceptable in both 2.2—supporting understanding/message in, and 2.3—supporting expression/message out)*<br><br>***Example response set + scoring***<br>• Check understanding after each small chunk of information *(1 mark, giving benefit of the doubt that 'understanding' relates to the person interacting with the PWA—as per point above).*<br>• Use alternate or contrasting options to check understanding. *(1 mark [links to forced/fixed choice and yes/no questions], giving benefit of the doubt that 'understanding' relates to the person interacting with the PWA—as per point above).*<br>• Ask them how they like to express themselves – whether it be with pen and paper, drawings, or pictures. *(1 mark [while already provided as a response for 2.1 acknowledge competence, can give the mark twice as it also applies here])*<br>• Provide encouragement when they are having difficulty *(0 marks as fits better in 2.1 acknowledge competence)* and be patient *(1 mark, links to response relating to "give them time to respond").*<br><br>*(Total 4 of 4 marks awarded)*
